# Variation in Expression of Inflammation-Related Signaling Molecules with Profibrotic and Antifibrotic Effects in Cutaneous and Oral Mucosa Scars

**DOI:** 10.1155/2018/5196023

**Published:** 2018-11-28

**Authors:** Mihai Bucur, Octavian Dinca, Cristian Vladan, Cristiana Popp, Luciana Nichita, Mirela Cioplea, Patricia Stînga, Petronel Mustatea, Sabina Zurac, Ecaterina Ionescu

**Affiliations:** ^1^“Carol Davila” University of Medicine and Pharmacy, 37 Dionisie Lupu, 020021 Bucharest, Romania; ^2^Department of OroMaxilloFacial Surgery, Clinical Hospital of OroMaxilloFacial Surgery Prof. Dr. Dan Theodorescu, 17 Calea Plevnei, 010221 Bucharest, Romania; ^3^Department of Pathology, Colentina University Hospital, 21 Stefan cel Mare, 020125 Bucharest, Romania; ^4^Department of Surgery, Clinical Hospital “Dr. Ion Cantacuzino”, 5 Ioan Movila, 020475 Bucharest, Romania; ^5^Ambulatory of Orthodontics, Clinical Hospital of OroMaxilloFacial Surgery Prof. Dr. Dan Theodorescu, 17 Calea Plevnei, 010221 Bucharest, Romania

## Abstract

Wound healing is a complex biologic process evolving in three phases: inflammation, proliferation, and tissue remodeling controlled by numerous growth factors and cytokines. Oral mucosa wounds heal with significantly less important scars with less numerous macrophages and mast cells and more numerous myofibroblasts than cutaneous counterparts. We analyzed 32 cutaneous and 32 oral mucosa scars for TGFbeta1, TGFbeta2, TGFbeta3, TNFalpha, PDGF BB and FGF1 expression in mesenchymal cells, endothelial cells, macrophages, and multinucleated giant cells. We identified differences in the expression of profibrotic and antifibrotic factors in oral mucosa and skin scars; TGFbeta2 was positive in cutaneous multinucleated giant cells, TNFalpha was positive in cutaneous macrophages, and both were negative in oral mucosa while TGFbeta3 was positive in oral macrophages and mostly negative in cutaneous ones. PDGF BB and FGF1 were positive in oral endothelial cells and oral macrophages and negative in macrophages with opposite positivity pattern in cutaneous scars. Based on these findings, macrophage seems to be the key player in modulating pro- and antifibrotic processes in wound regeneration.

## 1. Introduction

Wound healing is a complex biologic process evolving in three phases: inflammation, proliferation, and tissue remodeling [1]. These phases are controlled by myriad of growth factors and cytokines and, to some extent, overlap in time. *Inflammation* occurs almost immediately after wound formation—after the blood coagulation and lasts 2–4 days; several cells are chemotactically attracted to the wound site—neutrophils (they engulf bacteria and foreign bodies) and monocytes (they differentiate into macrophages that phagocyte necrotic debris, neutrophils, and foreign bodies); later on, macrophages secrete growth factors that stimulate the formation of granulation tissue [2–4]. *Proliferation* includes several processes such as angiogenesis and fibroblasts proliferation and differentiation towards myofibroblasts (formation of granulation tissue) and epithelial cells proliferation (reepithelization) [5–9]. *Tissue remodeling* (contraction phase, “maturation”) is the last and the longest phase of tissue healing; it consists of collagen synthesis and degradation in order to align the newly formed collagen bundles along tension lines [10–12].

Immune system intervenes in the regeneration of different processes such as inflammation and debris clearance in the first stages and proliferation and differentiation of the stem cells in further steps [13, 14]. Macrophages present two functional variants: M1 (“proinflammatory” macrophages with high interleukin- (IL-) 12, low IL-10, no IL-13*α*1, and MS4A4A production) and M2 (“anti-inflammatory” macrophages with IL-13*α*1, MS4A4A, high IL-10, and low IL-12 production) [15, 16]. Both types of macrophages intervene in regeneration in different moments and have to be “switched on/off” accordingly—M1 macrophages have to be active in the beginning of the process (mainly local debris clearance and subsequent cytokines secretion—IL6, TNFalpha, IL1beta, and G-CSF stimulate skeletal muscle proliferation), while M2 macrophages are involved in tissue remodeling (in skeletal muscle lesion they promote myogenic differentiation) [17]; prolonged activity of M1 macrophages or too early activation of M2 macrophages will have negative effects on wound healing [13].

Lymphoid cells are involved in wound healing mainly by cytokine secretion. NK T cells and gamma-delta T cells intervene in liver regeneration, and T-reg cells are involved in muscle regeneration and in oligodendrocyte differentiation and myelin regeneration [18–20]. Mast cells intervene in wound healing by releasing several factors with diverse functions including cytokines (IL-1 and TNFalpha) and growth factors (TGFbeta1 or PDGF) and favor collagen deposition [21, 22]. They are denser in more mature mice embryos than in younger ones, in dermis than oral lamina propria in adult pigs, and in hypertrophic than normotrophic human scars [23–25]; also, drugs with effects of inhibiting mast cell degranulation determine reduced contraction in pigs' wounds [26]. All these arguments favor the hypothesis that mast cells are directly related to fibrotic processes.

From all these data, it is obvious that inflammation can have both positive and adverse effects on wound healing, possibly some data obtained in animals being irreproducible in humans. The study of wound healing in human is extremely difficult due to ethical issues that hamper systematic approach; animal models are also difficult to use due to significant differences in healing process between species; data gathered in small mammals (mice, rats, and/or rabbits) are not reproducible in humans; pigs, however, have similar cutaneous structure and relatively similar healing mechanisms [27]. Systematic studies in pigs and studies in humans reveal differences in wound healing between cutaneous lesions and lesions in the oral mucosa. Oral mucosa wounds heal with significantly less important scars, thus deciphering specific healing mechanisms will offer valuable lessons to be applied to prevent severe scarring [28].

Healing in the oral mucosa involves less numerous macrophages and mast cells and more numerous myofibroblasts; also, TGFbeta expression is reduced in oral mucosa lesions; diminished inflammation and reduced tissue remodeling (wound contraction) favor a scarless healing. Local microenvironment is especially important in regulating myofibroblastic function since oral mucosa lesions heal with less collagen deposition than cutaneous ones despite the increased number of myofibroblasts [29]. Local inflammatory response vary according to the moment of loading of dental implants [30]. Cytokines influence on oral mucosa healing may represent the rationales for using autolog platelet-rich fibrin concentrates to favor local repair [31].

Considering all these pro's and con's, we decide to study the expression of several growth factors and cytokines in cutaneous and oral mucosa scars in humans.

## 2. Material and Methods

We analyzed 32 cutaneous scars and 32 mucosal scars. The cases were selected from the archives of the Department of Pathology of Colentina University Hospital; cutaneous scars specimens were reexcisions for previously resected cutaneous tumors; some specimens presented residual tumors but fragments without tumors were selected for this study (at least 4 mm distance between the residual tumor and the area of the scar selected for analysis).

All the tissue fragments were routinely processed (fixation for 24–72 hours in 10% buffered formalin; washing for 1–2 hours in running tap water); automatic histopathologic processing on a Leica ASP200S tissue processor (90 min ethanol 70° at 40°C, 105 min ethanol 80° at 40°C, 105 min ethanol 96° at 40°C, 60 min ethanol 100° at 40°C, 90 min ethanol 100° at 40°C, 90 min ethanol 100° at 40°C, 2 hours xylene at 52°C 3 bath, 1 hour paraffin 58°C, 2 hours paraffin 58°C, and 3 hours paraffin 58°C). For paraffin embedding, we used a Thermo Fisher Microm EC 1150 H embedding station; 30 slides of 3 microns thick sections were cut with a Leica RM 2265 rotary microtome; routine stains (hematoxylin and eosin (HE)), special stains (periodic acid-Schiff (PAS)), and immunohistochemical (IHC) tests were performed.

IHC tests used several primary antibodies: TGFbeta1, TGFbeta2, TGFbeta3, TNFalpha, PDGF BB, and FGF1; source, clones, specific pretreatments, and dilutions are specified in Table 1. The IHC stains were performed on an automated immunostainer Leica Bond III using Bond TM polymer refine detection (with DAB chromogen) and Bond TM polymer refine red detection. All the stains with one antibody were performed in the same day (to minimize technique-induced variations) with one negative and positive external control for each antibody; negative control consisted of IHC stains without primary antibody; positive external controls consist of normal human spleen for TGFbeta1, TGFbeta2, and TGFbeta3, human breast cancer for TNFalpha, normal human pancreas for PDGF BB, and normal human kidney for FGF1.

Two independent pathologists examined the slides; a three-grade semiquantitative scale for positivity was used: 0, negative; 1, faint positive (positivity evident when slides were examined with 40x); 2, intense positive (positivity evident when slides were examined with 10x) no matter the number of positive cells; the positivity was recorded for mesenchymal cells (fibroblasts and fibrocytes), endothelial cells, macrophages, and, when present, multinucleated giant cells. The level of positivity was interpreted in correlation with gender, localization, presence of residual tumor, and age of the scar. The statistical analysis of data was performed using EXCEL and EPIINFO programs; the results were considered statistically significant for *P* (*χ*2) was lower than 0.05.

This study was approved by Colentina University Hospital Ethic Committee, and all patients included agreed to participate in research studies.

## 3. Results

The cases were included in two groups: group A for cutaneous scars and group B for oral mucosa scars.

### 3.1. General Data

**Figure 1:**
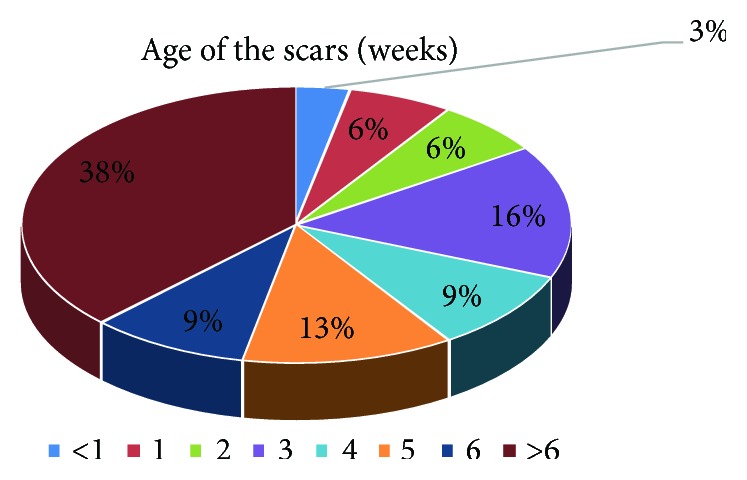
Age of the scars (weeks).

Group A included 32 patients, 13 males (40.62%) and 19 females (59.38%), between 8 and 79 years old (median 53 yrs, medium age 50.84 years). The scars were located on the head (5 cases, 15.62%), trunk (13 cases, 40.62%), limbs (10 cases, 31.25%; one case right arm and 9 cases inferior limbs), and special areas (4 cases, 12.50%; 3 cases from the skin of the breasts and one case from the axilla). The scars were as old as 3 to 504 days (Figure 1).

The scars were reexcised after previous resection of benign lesions (14 cases, 43.74%) or malignant tumor (18 cases, 56.25%; 13 melanomas, one basal cell carcinoma, 2 squamous cell carcinomas, one dermatofibrosarcoma protuberans, and one myxofibrosarcoma). Six cases (18.75%) had residual tumors (melanoma, squamous cell carcinoma, dermatofibrosarcoma protuberans, myxofibrosarcoma, melanocytic nevus, and capillary hemangioma), but, as we stated before, the area selected for analysis in our study was located at some distance from the residual tumor.

Group B (oral mucosa biopsies) included 32 patients; 11 patients were males (34.37%) and 21 females (65.62%), between 14 and 64 years old (median 22 yrs, medium age 25.19 years). All the biopsies originated from gingival areas.

All the cases presented granulation tissue or cicatricial collagen within the dermis/corion. In cutaneous scars, the inflammatory infiltrate consisted of lymphocytes and macrophages; 17 cases (53.12%) presented multinucleated giant cells, in some cases, in relation to translucid not structured material (suture material). In oral mucosa scars, the inflammatory infiltrate consisted of lymphocytes, macrophages, and, in frequent cases (18 cases, 56.25%), numerous plasma cells were present, occasionally with intracytoplasmic hyaline inclusions (Russell's bodies) as evidence of immunoglobulin production; no multinucleated giant cells were present in oral scars. Very scanty plasma cells were present in cutaneous scars.

### 3.2. Immunohistochemical Expression of Signaling Molecules in Cutaneous versus Oral Mucosa Scars (Data Are Summarized in Table 2)

#### 3.2.1. TGFbeta1, TGFbeta2, and TGFbeta3

**Figure 2:**
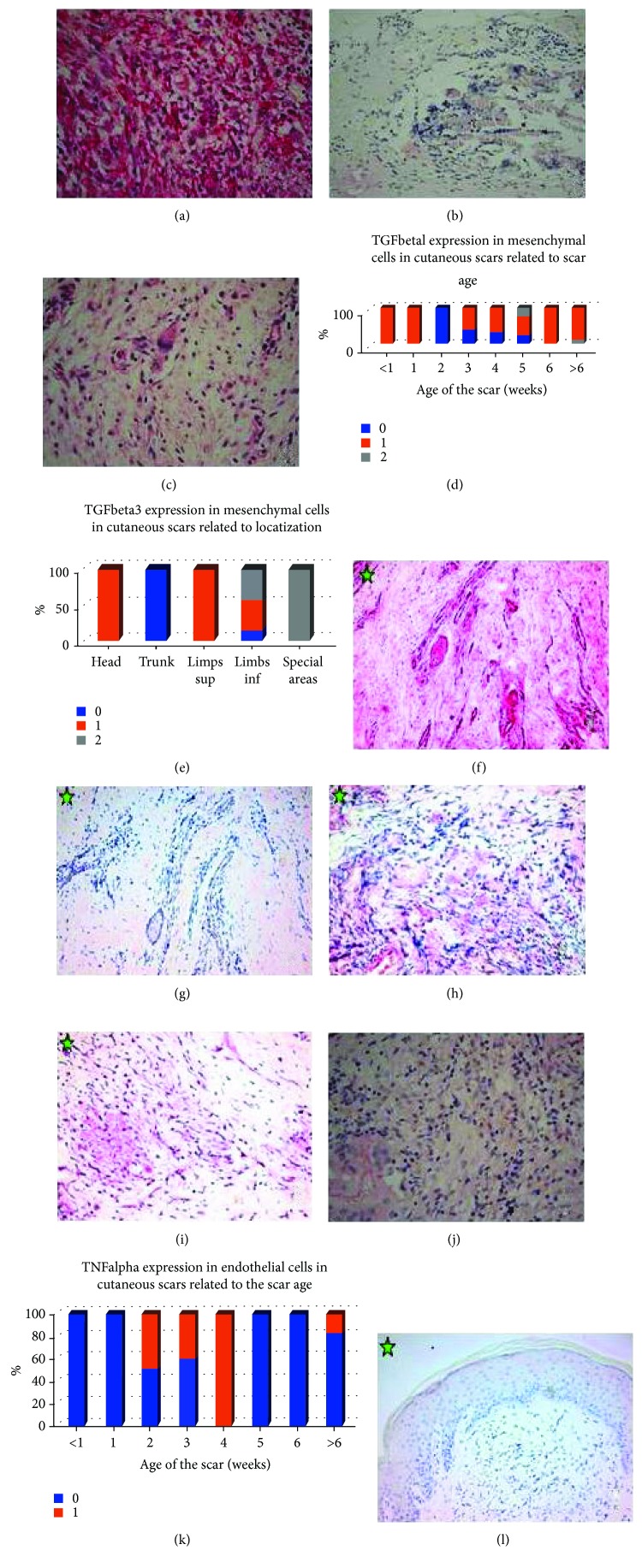
TGFbeta1, TGFbeta2, TGFbeta3, and TNFalpha expression. (a) Cutaneous scar. Intense positivity of TGFbeta1 (2+) in mesenchymal and endothelial cells. TGFbeta1 x400. (b) Cutaneous scar. Very faint positivity of TGFbeta2 in multinucleated giant cells engulfing foreign material (suture); macrophages, mesenchymal, and endothelial cells are negative. TGFbeta2 x200. (c) Cutaneous scar. TGFbeta3 intense positivity in macrophages, multinucleated giant cells, mesenchymal, and endothelial cells. TGFbeta3 x400. (d). TGFbeta1 expression in mesenchymal cells in cutaneous scars related to scar age. (e). TGFbeta3 expression in mesenchymal cells in cutaneous scars related to localization. (f). Oral mucosa scar. Intense positivity of TGFbeta1 (2+) in mesenchymal, endothelial cells, and macrophages. Small island of odontogenic epithelium also positive. TGFbeta1 x200. (g) Oral mucosa scar. Negativity for TGFbeta2. The island of odontogenic epithelium also negative TGFbeta2 x200. (h). Oral mucosa scar. Intense positivity of TGFbeta3 (2+) in endothelial cells and macrophages. TGFbeta3 x400. (i) Oral mucosa scar. Intense positivity of TGFbeta3 (2+) in endothelial cells and faint positivity in macrophages and multinucleated giant cells. TGFbeta3 x400. (j). Cutaneous scar. Faint positivity of TNFalpha in macrophages and multinucleated giant cells; mesenchymal and endothelial cells are negative. TNFalpha x400. (k) TNFalpha expression in endothelial cells in cutaneous scars related to the scar age. (l) Oral mucosa scar. Negativity for TNFalpha. TNFalpha x200.

TGFbeta1 was expressed mainly in endothelial cells (27 positive cases, 84.37%; 17 cases 1+ and 10 cases 2+) and mesenchymal cells (25 cases, 78.12%; 24 cases 1+ and one case 2+) and less frequently and fainter in macrophages (12 cases, 37.5%; all of them 1+ positive) and multinucleated giant cells (9 cases, 1+ of 17 cases with multinucleated giant cells) (Figure 2(a)).

TGFbeta2 was negative in mesenchymal cells, endothelial cells, and macrophages. Multinucleated giant cells were usually faint positive (13 cases, 1+ of 17 cases with multinucleated giant cells, 76.47%) (Figure 2(b)).

TGFbeta3 was expressed mainly in endothelial cells (26 positive cases, 81.25%; 12 cases 1+ and 14 cases 2+) and mesenchymal cells (23 cases, 71.87%, all faint positive 1+) and less frequently in macrophages (14 positive cases, 43.74%; 7 cases 1+ and 7 cases 2+) and multinucleated giant cells (10 positive cases, 58.82%; 5 cases 1+ and 5 cases 2+ of 17 cases with multinucleated giant cells) (Figure 2(c)).

In the case of TGFbeta1, the only statistical association was recorded for TGFbeta1 expression in mesenchymal cells in correlation with the age of the scar—we noticed a tendency towards lack and/or diminishing of TGFbeta1 expression in mesenchymal cells in the scars of 2–5 weeks compared with younger (less than 2 weeks) or older (more than 5 weeks) cutaneous scars—*P* = 0.05 (Figure 2(d)). There was a statistically significant correlation between TGFbeta3 expression in mesenchymal cells and localization of the scar—no scars on the trunk had TGFbeta3 expression in mesenchymal cells while all the scars from special areas had intense diffuse positivity—*P* < 0.001 (Figure 2(e)).

In oral mucosa biopsies, TGFbeta1 was expressed in all the cases in mesenchymal cells, endothelial cells, macrophages, and plasma cells in all cases with obvious positivity in low power (2+) in almost all the cells (Figure 2(h)). TGFbeta2 was negative in all the cases (Figure 2(i)). TGFbeta3 was negative in mesenchymal cells and intensely diffuse positivity (2+) in endothelial cells and plasma cells; all the cases had TGFbeta3 positivity in macrophages, half of them (50.00%) being 1+, while the other half were intense diffuse positive (2+) (Figures 2(j)–2(k)).

#### 3.2.2. TNFalpha

TNFalpha expression was faint in each type of cells we investigated (1+). Most frequent macrophages and macrophage-derived cells showed TNFalpha positivity (14 cases, 43.74% showed TNFalpha faint positivity in macrophages and 12 cases of 17, 70.58% showed similar positivity in multinucleated giant cells); less numerous cases had mesenchymal cells positivity (15 cases, 46.875%) and endothelial cells positivity (8 cases, 25.00%) (Figure 2(f)). We identified a statistically significant association between TNFalpha expression in endothelial cells and the age of the scar—scars 2 to 4 weeks old tend to express TNFalpha compared with very recent scars or older ones—*P* = 0.036 (Figure 2(g)). Interestingly, in week 4, all the cases showed TNFalpha positivity within endothelial cells (all male patients, scar located on the head, trunk, and axilla, resections after melanoma or basal cell carcinoma, no residual tumor present in either case).

In oral mucosa biopsies, TNFalpha was negative in all the cases in mesenchymal cells, endothelial cells, and macrophages; cases with plasma cells within the inflammatory infiltrate showed 1+ or 2+ positivity in plasma cells (Figure 2(l)).

#### 3.2.3. PDGF BB

**Figure 3:**
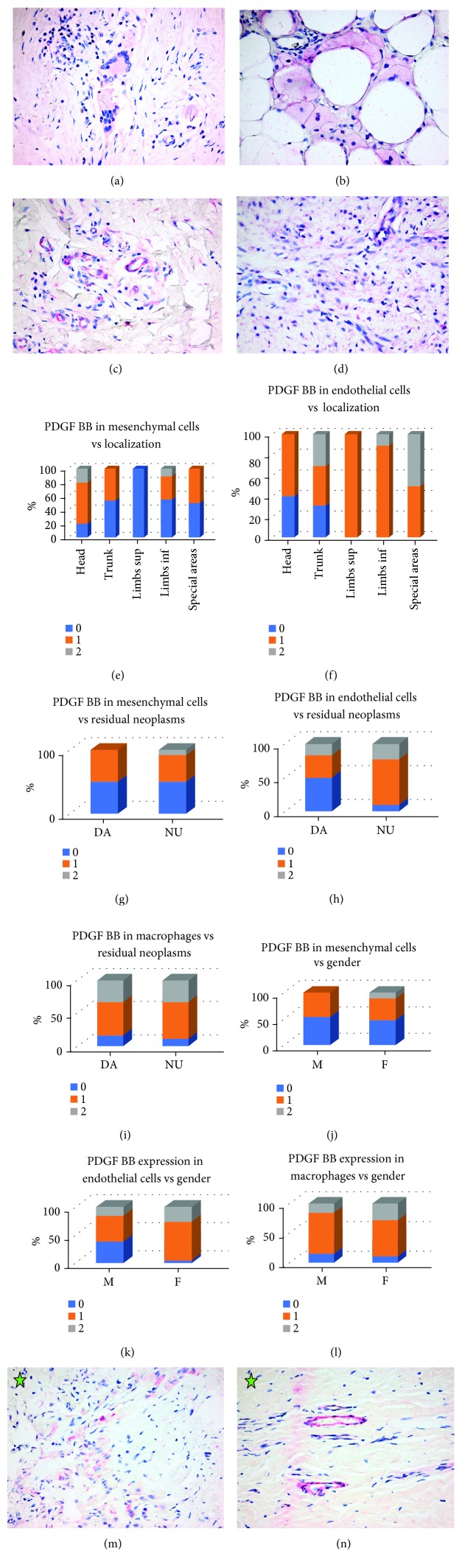
PDGF BB expression. (a) Faint positivity of PDGF BB (1+) in multinucleated giant cells. Cutaneous scar. PDGF BB x400. (b) Faint positivity of PDGF BB (1+) in macrophages. Cutaneous scar. PDGF BB x400. (c) Intense positivity of PDGF BB (2+) in mesenchymal and endothelial cells. Cutaneous scar. PDGF BB x400. (d). Faint positivity of PDGF BB (1+) in mesenchymal and endothelial cells. Cutaneous scar. PDGF BB x400. (e) PDGF BB expression in mesenchymal cells in cutaneous scars related to localization. (f) PDGF BB expression in endothelial cells in cutaneous scars related to localization. (g) PDGF BB expression in mesenchymal cells in cutaneous scars related to presence of residual neoplasms. (h) PDGF BB expression in endothelial cells in cutaneous scars related to presence of residual neoplasms. (i) PDGF BB expression in macrophages in cutaneous scars related to the presence of residual neoplasms. (j) PDGF BB expression in mesenchymal cells in cutaneous scars related to patient's gender. (k) PDGF BB expression in endothelial cells in cutaneous scars related to patient's gender. (l) PDGF BB expression in macrophages in cutaneous scars related to patient's gender. (m) Oral mucosa scar. Intense positivity of PDGF BB (2+) in macrophages. PDGF BB x400. (n) Oral mucosa scar. Intense positivity of PDGF BB (2+) in endothelial cells. PDGF BB x400.

PDGF BB was expressed mostly in macrophages and multinucleated giant cells (macrophages; 28 cases, 87.50% (21 cases 1+ and 7 cases 2+) and multinucleated giant cells; 16 of 17 cases, 94.11% (12 cases 1+ and 4 cases 2+)) and also in endothelial cells in 26 cases, 81.25% (19 cases 1+ and 7 cases 2+) and mesenchymal cells in 16 cases, 50.00% (14 cases 1+ and 2 cases 2+) (Figures 3(a)–3(d)). There was a tendency towards PDGF BB overexpression in mesenchymal cells in scars located in the head area (*P* < 0,001) (Figure 3(e)) and PDGF BB overexpression in endothelial cells in scars located in the limbs and special areas (*P* = 0, 05) (Figure 3(f)); more powerful statistically significant figures were obtained when PDGF BB overexpression in endothelial cells in scars located in the limbs or special areas were compared with PDGF BB expression in same cells in scars originating from the head or trunk (P trunk vs. limbs 0.012; P trunk vs. special areas <0.001; P head vs. limbs 0.002; P head vs. special areas 0.0001) (Figure 3(f)).

Also, PDGF BB was overexpressed in all types of cells (mesenchymal cells *P* = 0.009, endothelial cells *P* = 0.001, and macrophages *P* = 0.0001) in scars with no residual tumor (Figure 3(g)–3(i)) and in scars in female patients (mesenchymal cells *P* = 0014, endothelial cells *P* < 0.001, and macrophages *P* < 0.0001) (Figures 3(j)–3(l)). Interestingly, PDGF BB overexpression was noted in multinucleated giant cells in a scar with residual tumors and also in scars in male patients (in both circumstances *P* < 0.001); we looked in the group of scars with multinucleated giant cells that showed a female predominance compared with the general data of group A (70.58% females in scars with multinucleated giant cells compared with 59.38% in group A) and more numerous cases with residual tumor (23.53% in scars with multinucleated giant cells compared with 18.75% in group A), thus explaining the differences with PDGF BB expression in macrophages. In oral biopsies, PDGF BB was positive 2+ in all cases in endothelial cells, macrophages, and also in plasma cells when present; oral mesenchymal cells were negative for PDGF BB (Figures 3(m)–3(n)).

#### 3.2.4. FGF1

**Figure 4:**
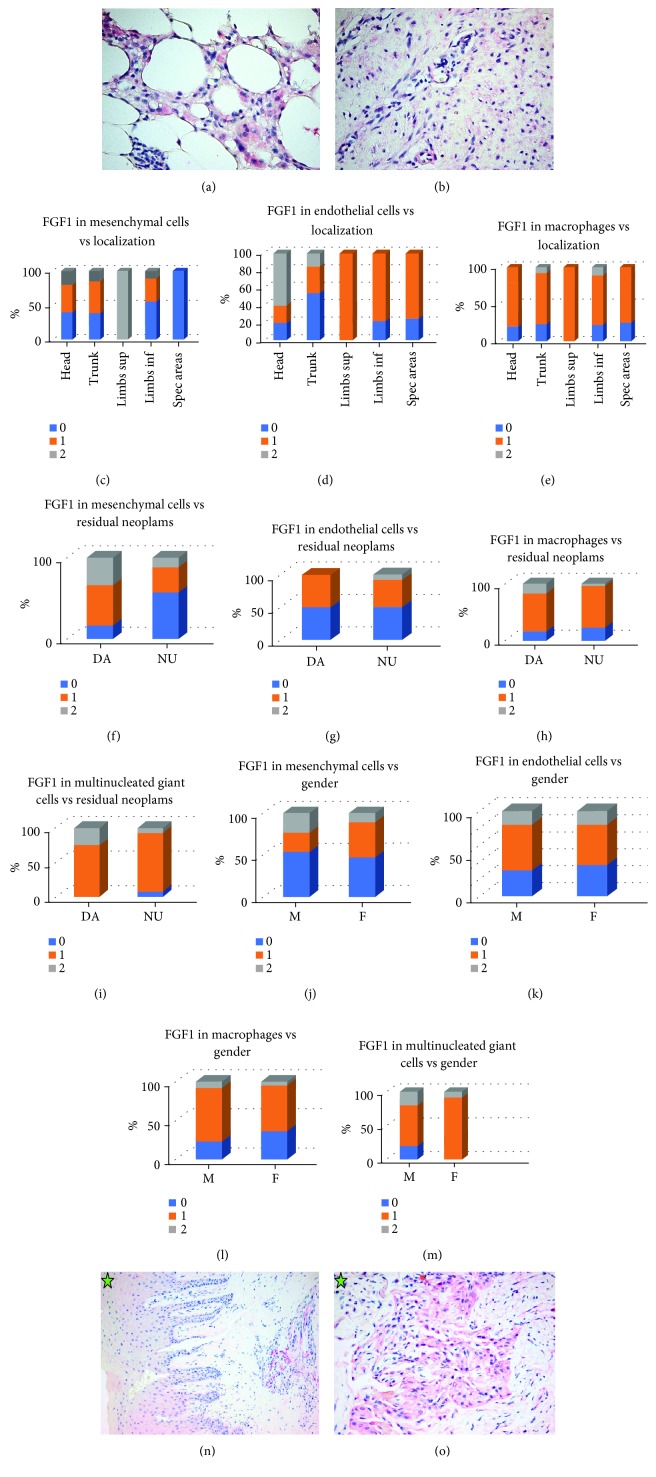
FGF1 expression. (a) Faint positivity of FGF1 (1+) in macrophages and multinucleated giant cells. Cutaneous scar. FGF1 x400. (b) Faint positivity of FGF1 (1+) in mesenchymal and endothelial cells. Cutaneous scar. FGF1 x400. (c) FGF1 expression in mesenchymal cells in cutaneous scars related to localization. (d) FGF1 expression in endothelial cells in cutaneous scars related to localization. (e) FGF1 expression in macrophages in cutaneous scars related to localization. (f) FGF1 expression in mesenchymal cells in cutaneous scars related to the presence of residual neoplasms. (g) FGF1 expression in endothelial cells in cutaneous scars related to the presence of residual neoplasms. (h) FGF1 expression in macrophages in cutaneous scars related to the presence of residual neoplasms. (i) FGF1 expression in multinucleated giant cells in cutaneous scars related to the presence of residual neoplasms. (j) FGF1 expression in mesenchymal cells in cutaneous scars related to patient's gender. (k) FGF1 expression in endothelial cells in cutaneous scars related to patient's gender. (l) FGF1 expression in macrophages in cutaneous scars related to patient's gender. (m) FGF1 expression in multinucleated giant cells in cutaneous scars related to patient's gender. (n) Oral mucosa scar. Intense positivity of FGF1 (2+) in endothelial cells. Hyperplasia of rete ridges specific to alveolar mucosa. FGF1 x200. (o) Oral mucosa scar. Faint positivity of FGF1 (1+) in macrophages. FGF1 x400.

FGF1 expression was noted in half of the cases (50.00%) in mesenchymal cells (11 cases 1+ and 5 cases 2+), two-thirds (68.75%) in endothelial cells (16 cases 1+ and 5 cases 2+), and almost three quarters of cases (78.12%) in macrophages (23 cases 1+ and 2 cases 2+). All but one case (94.11%) showed FGF1 positivity in multinucleated giant cells (14 cases 1+ and 2 cases 2+) (Figures 4(a)–4(b)). We looked for FGF1 expression related to location. There was a statistically significant overexpression in the mesenchymal cells of scars from the limbs compared with the mesenchymal cells of scars from special areas (*P* < 0.001) and in the endothelial cells of scars from the head and trunk compared with the limbs and special areas (*P* = 0.05). Macrophages expressed more intense FGF1 in scars from the trunk or limbs compared with the head (*P* = 0.007 and *P* = 0.0005, respectively); also, they expressed FGF1 more often in scars from the limbs or special areas compared with the trunk or in scars from the limbs compared with special areas (*P* < 0.001 in all circumstances) (Figures 4(c)–4(e)). Moreover, FGF1 was overexpressed in all types of cells (mesenchymal cells, endothelial cells, macrophages, and multinucleated giant cells) in cases with residual neoplasm present in the vicinity of the scar tissue we examined (*P* = 0.008, *P* = 0.012, *P* = 0.002, *P* < 0.001, respectively) (Figures 3(f)–3(i)) and in female patients (*P* = 0.009, *P* = 0.009, *P* = 0.001, *P* < 0.001, respectively) (Figures 4(j)–4(m)). FGF1 expression in oral mucosa scars had similar positivity as PDGF BB: positive 2+ in all cases in endothelial cells, positive 1+ in macrophages, and negative in mesenchymal cells (Figures 4(n)–4(o)).

## 4. Discussions

We studied several growth factors expression in cutaneous and oral mucosa humans scars: TGFbeta1, TGFbeta2, TGFbeta 3, TNFalpha, PDGF BB, and FGF1.

### 4.1. TGFbeta1, TGFbeta2, and TGFbeta3

Our data showed the expression of TGFbeta1 (profibrotic growth factor) in all cases in oral specimens and numerous cases in skin fragments; however, there is an obvious relation to the age of the cutaneous scars, the young and old ones having less if any TGFbeta1 expression. TGFbeta2 (the other profibrotic factor) was positive in more than ¾ of the cases of skin specimens in multinucleated giant cells and absent in oral specimens. TGFbeta3 (with probable antifibrotic effects) was positive in all oral specimens in macrophages either intense or mild positivity, while less than half of the cutaneous fragments showed macrophagic positivity.

TGFbeta1, TGFbeta2, and TGFbeta3 belong to the TGFbeta/activin/Nodal subfamily of TGFbeta superfamily [32]. They are involved in numerous biological processes such as cellular differentiation, cell migration, apoptosis, cell-cycle arrest, production of extracellular matrix, epithelial to mesenchymal transition, or wound healing [33–35]. The overexpression of TGFbeta determines pathologic tissue fibrosis. TGFbeta1 is upregulated in early granulation tissue thus determining an increase in number and activity of SMA positive myofibroblasts thus stimulating neovascularization, collagen deposition, and wound contraction; all these processes are responsible for scar formation in adulthood. Wound in embryos predominantly express TGFbeta3 and to a significantly lesser extent, TGFbeta1; moreover, adding TGFbeta3 to a wound in adult tissues (or neutralizing TGFbeta1 and TGFbeta2) determines less or none scarring [36–42].

It is known that the major source of TGFbeta in a wound is represented by platelets; platelets release TGFbeta from their secretory granules in inactive form; part of it will be immediately activated (in the first moments of wound appearance) by trombospondin-1 and released together with TGFbeta from platelets secretory granules and also some time later by plasmin which disintegrates blood clot [43, 44]. The second wave of TGFbeta activation occurs after macrophages also occupy the wound territory via plasmin (macrophages secrete plasminogen activators) [45, 46].

TGFbeta is involved in all phases of wound healing. In the first phase (inflammation), TGFbeta are present from the very beginning. They are supplied in their inactive forms by platelets and are activated by thrombospondin1 and plasmin; in this stage, they act as chemotactic cytokines for neutrophils, monocytes, and macrophages [43, 47]. Later on, activated macrophages (local cells or differentiated from recruited circulating monocytes) release supplementary quantities of TGFbeta that stimulates angiogenesis, fibroblasts chemotaxis, and proliferation in second phase (proliferation, “granulation tissue formation”) [48]. TGFbeta stimulates the differentiation of fibroblasts into myofibroblasts [40, 49] and stimulates the production of components of extracellular matrix (ECM): collagen, fibronectin, and fibronectin receptor [50]; also, by inhibiting both production and activity of matrix metalloproteinases (MMPs) and stimulating the expression of tissue inhibitors of matrix metalloproteinases (TIMPs), they determine a reduction of ECM components degradation [5]. TGFbeta activity in angiogenesis involves all aspects of the process; they promote endothelial cell migration and differentiation and also tubule formation [7].

In maturation phase, TGFbeta intervenes in a delicate balance between MMPs and TIMPs activation and also in increasing lysyl oxidases responsible of cross-linking of collagen with subsequent increase of tensile strength of the wound [51]. Studies performed on animals showed that TGFbeta1 occurs in the wound almost immediately after wound occurrence (5 minutes), then TGFbeta2 and TGFbeta3 occur, surpassing by far TGFbeta1 levels at 24 hours after wound occurrence; there is another peak of TGFbeta1 at 5 days after wound occurrence [52–54]. Also, in embryos, wounds express mainly TGFbeta3 (both in epithelial cells and fibroblasts), while TGFbeta1 and TGFbeta2 have very low levels of expression; in adults, wounds express mainly TGFbeta1 and TGFbeta2. Since wound in embryo heal without scarring while those in adults do not, it is a sound presumption that TGFbeta3 favor scar-free healing opening the gates for new therapies [55, 56].

### 4.2. TNFalpha

In our study, TNFalpha was negative in all cells but positive in plasma cells in oral specimens, while almost half of the cutaneous specimens showed TNFalpha positivity in macrophages; also, some skin cells (both mesenchymal and endothelial cells) had some positivity for TNFalpha also with a tendency towards expression in not so young or old scars. In week 4, all the patients (males, head, trunk, or axilla scars, resection after tumor without residual tumor present) showed TNFalpha expression in endothelial cells.

TNFalpha enhances the inflammation in wound repair, hence contributing to an impaired healing both in chronic and acute wounds. Studies revealed and increased TNFalpha levels (locally, in the wound territory, and systemically) in otherwise healthy elderly patients with active chronic venous ulcers. Also, in animals, experiments with secretory leukocyte protease inhibitor (SLPI) null mice showed that wound healing in these animals is deficient, and TNFalpha is increased in wound area (demonstrated both by RT-PCR and immunohistochemically in local inflammatory cells and epithelia); moreover, local TNFalpha inhibition with anti-TNFalpha antibody accelerates the rate of healing in a dose-dependent manner in SLPI null mice and also improve healing in wild-type mice used as controls [57].

TNFalpha induces the expression of MMP2 and MMP9 and inhibits the local accumulation of fibroblasts either by direct inhibition of chemoattraction or by attracting an impressive number of inflammatory cells in the wound area; both these actions diminish the collagen deposition and impair the wound healing [58, 59].

### 4.3. PDGF BB

Our study revealed PDGF BB positivity in all oral specimens in endothelial cells and macrophages but not in mesenchymal cells. In skin scars fragments, there were some expression in mesenchymal cells (half of the scars) with statistic association with feminine gender, head localization, and absence of residual tumor. Few cases of cutaneous scars had no PDGF BB expression in endothelial cells (18.75%) or macrophages (12.5%).

PDGF is a dimeric glycoprotein with mitogenic effects on mesenchymal cells such as fibroblasts, osteoblasts, and smooth muscle cells; also, it is implicated in angiogenesis and fibrosis. Two types of subunits are identified A and B, three variants of PDGF being thus possible: PDGF-AA, PDGF-BB, and PDGF-AB [60]. PDGF stimulates reepithelialization, revascularization, and complete wound closure in ischemic skin and hyperglicemic mice, mainly due to its mitogenic effects on both keratinocytes and endothelial cells [61]. The association of PDGF-BB with TGF-alpha in topic application on wounds in genetically diabetic (C57BL/KsJ-db/db) mice induces the acceleration of healing to a level almost similar to nondiabetic mice [62]. The treatment of leg ulcers in diabetic patients with becaplermin gel (a recombinant human PDGF approved for topical applications) stimulates complete healing and shortens the time to healing [63]. However, caution should be exerted in becaplermin treatment due to the increased risk of both local infections (infected skin ulcer, cellulitis, and osteomyelitis) and death due to malignancies, currently the drug being no longer authorized [64].

### 4.4. FGF1

FGF1 expression was noted in our study in similar manner as PDGF BB—oral scars lack FGF1 in mesenchymal cells, while 50% of the skin scars had FGF1 mesenchymal cells positivity, statistically associated with the limbs location and residual tumor present in the vicinity of the scar. More numerous cases of skin scars lack FGF1 expression in endothelial cells and/or macrophages than PDGF BB, but all oral scars show FGF1 expression in these types of cells.

Fibroblast growth factor (FGF) family includes 22 members designated by numbers from FGF1 to FGF23 (FGF15 is lacking in humans). FGF1 (acidic FGF) has a large variety of functions; the most important ones being related to angiogenesis. It induces proliferation of endothelial cells with subsequent organization in tubes and formation of new vessels in myocardium [65]; its function as angiogenic factor overcomes that of consecrated angiogenic factors as vascular endothelial growth factor (VEGF) or platelet-derived growth factor (PDGF) [66]. Based on its angiogenic function, FGF1 is a major player in wound healing [67, 68]; it has decreased gene expression in early phases of wound healing in diabetic patients [69], and it has a proven effect on reepitelization in NONcNZO10/LtJ mouse (model for impaired wound healing in type 2 diabetes) [70]. Also, FGF1 is involved in bone regeneration, and it is a promising biomolecule to be used in humans [71].

## 5. Conclusions

We identified the differences in the expression of profibrotic and antifibrotic factors in oral mucosa and skin scars; TGFbeta2 was positive in cutaneous multinucleated giant cells, TNFalpha was positive in cutaneous macrophages, and both were negative in oral mucosa while TGFbeta3 was positive in oral macrophages and mostly negative in cutaneous ones. PDGF BB and FGF1 were positive in oral endothelial cells and oral macrophages and negative in macrophages with opposite positivity pattern in cutaneous scars. Based on these findings, macrophage seems to be the key player in modulating pro- and antifibrotic processes in wound regeneration. Further studies are needed in order to establish the mechanisms favoring scarless healing and subsequent application in daily practice.

## Figures and Tables

**Table 1:** Primary antibodies: technical specifications.

Primary antibody	Source	Clone	Epitope retrieval^∗^	Dilution	Incubation period (min)
TGFbeta1	ABCAM	Policlonal	HIER pH 6	8/200	60
TGFbeta2	ABCAM	ab36495	HIER pH 6	0.5/250	60
TGFbeta3	ABCAM	Policlonal	HIER pH 6	8/200	60
TNFalpha	ABCAM	Policlonal	HIER pH 6	1/250	60
PDGF BB	ABCAM	Policlonal	HIER pH 6	1/250	30
FGF1	ABCAM	Policlonal	HIER pH 6	1/200	60

^∗^HIER = heat-induced epitope retrieval.

**Table 2:** Comparative summary of the differences between the findings of the expression of signaling molecules in cutaneous versus oral mucosa scars.

Type of cells	Cutaneous scars	Oral mucosa scars
*TGFbeta1*		
Mesenchymal cells	74.99% 1+	100% 2+
	3.12% 2+	
Endothelial cells	53.12% 1+	100% 2+
	31.25 2+	
Macrophages	37.5% 1+	100% 2+
Multinucleated giant cells	52.94% 1+	Not present
Plasma cells	Not present	56.25% 2+
*TGFbeta2*		
Mesenchymal cells	Negative	Negative
Endothelial cells	Negative	Negative
Macrophages	Negative	Negative
Multinucleated giant cells	76.47% 1+	Not present
Plasma cells	Not present	Negative
*TGFbeta3*		
Mesenchymal cells	71.87% 1+	Negative
Endothelial cells	37.5% 1+	100% 2+
	43.75% 2+	
Macrophages	21.87% 1+	50.00% 1+
	21.87% 2+	50.00% 2+
Multinucleated giant cells	29.41% 1+	Not present
	29.41% 2+	
Plasma cells	Not present	56.25% 2+
*TNFalpha*		
Mesenchymal cells	46.87% 1+	Negative
Endothelial cells	25.00% 1+	Negative
Macrophages	43.74% 1+	Negative
Multinucleated giant cells	70.58% 1+	Not present
Plasma cells	Not present	28.12% 1+
		28.12% 2+
*PDGF BB*		
Mesenchymal cells	43.75% 1+	Negative
	6.25% 2+	
Endothelial cells	59.36% 1+	100% 2+
	21.87 2+	
Macrophages	65.63% 1+	100% 2+
	21.87% 2+	
Multinucleated giant cells	70.58% 1+	Not present
	23.53% 2+	
Plasma cells	Not present	56.25% 2+
*FGF1*		
Mesenchymal cells	34.37% 1+	Negative
	15.62% 2+	
Endothelial cells	52.38% 1+	100% 2+
	16.37% 2+	
Macrophages	71.87% 1+	100% 1+
	6.25% 2+	
Multinucleated giant cells	82.34% 1+	Not present
	11.76% 2+	
Plasma cells	Not present	56.25% 1+

## Data Availability

The datasets used and/or analyzed during the present study are included within the article; if supplemental information is needed, it is available from the corresponding author upon request except confidential data to whom access is restricted in order to protect patients' privacy.
